# Supplementary figures and images for: Evidence of pyrethroid resistance in Anopheles amharicus and Anopheles arabiensis from Arjo-Didessa irrigation scheme, Ethiopia
Source: PLoS One. 2022 Jan 14;17(1):e0261713. doi: 10.1371/journal.pone.0261713 (PMC8759678; doi:10.1371/journal.pone.0261713)

X 1 2 3 4 5 6 7 8 9 10 11 12 13

X X X X X X X X X X X X X

X 1 2 3 4 5 6 7 8 9 10 11 12 13

X X X X X X X X X X X X X

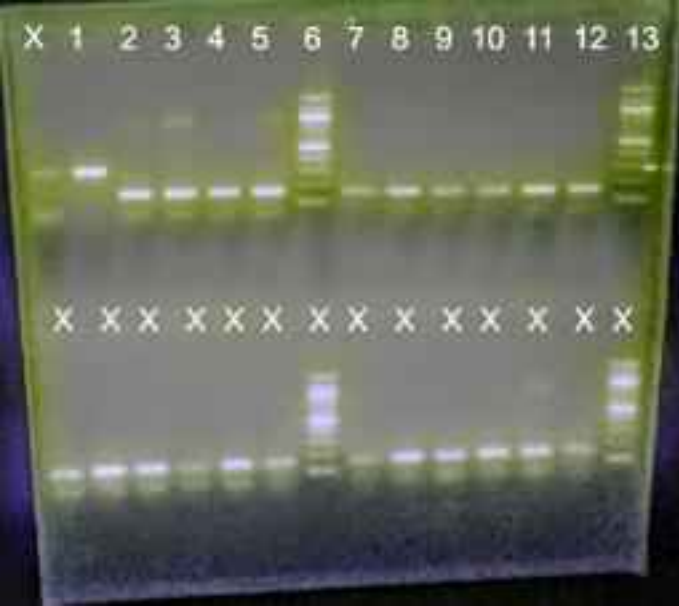

Supplement: S1 Raw images — (PDF) [file pone.0261713.s001.pdf]
