# Supplementary material for: Evidence of pyrethroid resistance in Anopheles amharicus and Anopheles arabiensis from Arjo-Didessa irrigation scheme, Ethiopia
Source: PLoS One. 2022 Jan 14;17(1):e0261713. doi: 10.1371/journal.pone.0261713 (PMC8759678; doi:10.1371/journal.pone.0261713)
Supplement: Attachment — Submitted filename: Review Remarks Githeko Ak.docx [file pone.0261713.s002.docx]

Review Remarks

Evidence of pyrethroid resistance in Anopheles amharicus and Anopheles arabiensis

from Arjo-Didessa irrigation scheme, Ethiopia

**General remarks**

The manuscript addresses an important vector control operational issues. The data generated will be useful to the Ethiopian National Malaria program.

It would be useful to provide some information on the relative importance of *An. amharicu*s as a vector relative to *An. arbiensis.*

Indicate which of the two species is more anthropophilic and thus more likely to come into contact with insecticides indoors.

**Specific remarks**

Anopheles amharicus was predominant species for every type of insecticide tested followed by

An. arabiensis (Table 1). The proportions of An. amharicus tested for propoxur was 100%

(n=26/26), for pirmiphos-methyl 90% (n=28/31) and for clothianidin 91.5% (n=43/47). The

distribution of An. amharicus and An. arabiensis tested again..

*Is the above sentence referring to proportion* ***mortality*** *? Please clarify.*

KT50 and KT90 values

*Spell out KT in full in the subheading.*

Previous study from the same area also revealed the co-existence of

these two sibling species in the sugarcane irrigation settings although *An. arabiensis* was the

predominating species [18.

*Could the species abundance in this and in the previous studies have been affected by seasonality ? An gambiae and An. arabiensis abundance in sympatric populations, is influenced by seasonality elsewhere in Africa.*

Long term use of public health insecticides and agricultural chemicals (pesticides, herbicides and

fertilizers) might have contributed for the resistance of An. arabiensis and An. amharicus against

pyrethroids in the study area.

Is there a history of the use of DDT for vector control in the study area ?. This could explain the cross resistance to pyrethroides.

Please provide any evidence that herbicides and fertilisers are linked to insecticide resistance and in particular to pyrethroides. The authors need to discuss the mechanisms involved. The studies in west Africa (Côte d’ Ivoire ) and Tanzania did not provide the mechanism involved.

Indicate the class of insecticides for clothianidin after its first mention : **neonicotinoid**

Similar studies in Ethiopia [32] and Africa [52] reported clothianidin susceptibility of malaria vectors.

From its efficacy, clothianidin is being highly recommend as viable candidate to replace IRS for...

It is nor IRS that is replaced, rather it is the insecticide such as DDT, deltamethrin, etc that are being replaced.
